# Benefits from ^18^F-FDG PET-CT-Based Radiotherapy Planning in Stage III Non-Small-Cell Lung Cancer: A Prospective Single-Center Study

**DOI:** 10.3390/cancers17121969

**Published:** 2025-06-13

**Authors:** Admir Mulita, Pipitsa Valsamaki, Eleni Bekou, Stavros Anevlavis, Christos Nanos, Athanasios Zisimopoulos, Alexandra Giatromanolaki, Michael I. Koukourakis

**Affiliations:** 1 Department of Radiotherapy/Oncology, School of Medicine, Democritus University of Thrace, 68100 Alexandroupolis, Greece; admirm@hotmail.gr (A.M.);; 2 Department of Nuclear Medicine, School of Medicine, Democritus University of Thrace, 68100 Alexandroupolis, Greece; pivalsam@med.duth.gr (P.V.); azisimop@med.duth.gr (A.Z.); 3 Medical Physics Laboratory, School of Medicine, Democritus University of Thrace, 68100 Alexandroupolis, Greece; ebekou@med.duth.gr; 4 Department of Pneumonology, Medical School, Democritus University of Thrace, 68100 Alexandroupolis, Greece; sanevlav@med.duth.gr; 5 Department of Pathology, School of Medicine, Democritus University of Thrace, 68100 Alexandroupolis, Greece; agiatrom@med.duth.gr

**Keywords:** non-small-cell lung cancer, radiotherapy, pet-ct

## Abstract

**Background/Objectives:** Lung cancer is the leading cause of cancer-related mortality worldwide. Accurate radiotherapy (RT) planning alongside chemotherapy and immunotherapy is critical for improving treatment outcomes for inoperable non-metastatic cases. Conventional computed tomography (CT)-based planning may be inadequate for accurately identifying tumor margins and the location of nodal disease. We investigated whether ^18^F-labeled fluorodeoxyglucose positron emission tomography (^18^F-FDG PET-CT) imaging can assist in target volume delineation for primary, nodal, and metastatic disease in the RT planning and overall therapeutic planning of patients. **Methods**: In this single-center, prospective study, we recruited 34 patients with histologically confirmed locally advanced non-small-cell lung carcinoma (NSCLC). All patients underwent ^18^F-FDG PET-CT-based RT simulation. Two sequential RT plans were created by the same radiation oncologist: one based on CT alone and the other PET-CT. Planning target volumes (PTVs) and PET-CT-guided adjustments were analyzed to assess their impact. Standardized protocols for immobilization, imaging, target delineation, and dose prescription were applied. **Results**: A total of 34 patients (31 males and 3 females) were recruited in the study. ^18^F-FDG PET-CT detected distant metastases in 7/34 (20.6%) patients, altering the overall therapeutic plan in 4/34 (11.8%) and allowing radical RT in 3 of them who had oligometastatic disease (8.8%). It modified RT planning in 26/34 (76.5%) patients and clarified malignancy in atelectatic areas. Nodal involvement was identified in 3/34 patients (8.8%) and excluded in 3/34 cases, avoiding unnecessary nodal irradiation. Additional involved nodes were revealed in 12/34 (35.3%) patients, requiring dose escalation. Overall, changes to the tumor PTV were made in 23/30 (76.6%) and to the nodal PTV in 19/30 (63.3%) cases (*p* < 0.0001). Primary tumor and nodal PTVs increased in 20/34 (66.7%) and 13/34 (43.3%), respectively. **Conclusions**: ^18^F-FDG PET-CT significantly improves RT planning by more precisely defining tumor and nodal volumes, identifying undetected lesions, and guiding dose adaptation. Larger long-term studies are required to confirm potential locoregional control and survival improvements.

## Simple Summary

This study aims to determine how ^18^F-FDG PET-CT functional imaging can enhance the accuracy of target volume delineation in patients undergoing radiotherapy for non-metastatic non-small-cell lung cancer, compared to traditional CT-based planning. By comparing sequential radiotherapy plans based on CT alone and PET-CT imaging, we noted important changes in the overall therapy plan due to the identification of metastatic and oligometastatic disease, and changes in the delineation of the tumor and nodal PTV due to increased precision in the detection of tumor margins and the documentation of involved nodal areas.

## 1. Introduction

Lung cancer is the dominant cause of cancer-related death worldwide among both genders [1]. In the GLOBOCAN database, provided by the International Agency for Research on Cancer (IARC), the estimated death toll was 1.8 million in 2022. The number of new cases is expected to increase twofold between 2022 and 2045 [2].

Lung cancer is mainly categorized into two main histological types: non-small-cell lung carcinoma (NSCLC), including adenocarcinoma, squamous cell carcinoma, and large-cell carcinoma, and small-cell lung carcinoma (SCLC). The collaboration between the World Health Organization (WHO), the National Comprehensive Cancer Network (NCCN), and the International Association for the Study of Lung Cancer (IASLC) developed international guidelines for more effective treatments and improved outcomes for lung cancer [1,3,4]. According to these guidelines, radiotherapy (RT) in combination with chemotherapy is the primary therapy for stage IIIb patients, and also for medically inoperable patients with lower-stage disease. During the past five years, adding maintenance immunotherapy after chemo-RT has become the gold standard, increasing the overall survival figures [5].

The locoregional control (LRC) offered by radiotherapy (RT) in patients with non-metastatic NSCLC is essential to achieving optimal disease-free survival rates and sustaining quality of life, even in patients with metastatic disease [6]. Volumetric modulated arc therapy (VMAT) combined with image-guided radiation therapy (IGRT) allows more accurate delineation of tumor and surrounding tissues and permits correction of position errors in the linear accelerator (LINAC). Moreover, it enables the adaptation of treatment plans based on tumor size changes during therapy. So, VMAT with IGRT increases accuracy and reduces unnecessary exposure of normal tissues to radiation treatment margins, allowing dose escalation and a better therapeutic index in lung cancer RT [7,8].

Target volumes in radiotherapy are typically delineated using computed tomography (CT) images. Lung lesions often overlap with atelectasis, making it challenging to distinguish the tumor margins, while small tumor deposits in the primary tumor margins or lymph nodes may easily escape the physician’s attention [9]. This may lead either to adopting larger target volumes that allow unwanted spread of radiation to healthy tissues or, in contrast, to omitting involved areas in the RT plan [10,11].

Functional imaging, such as ^18^Fluorine-labeled fluorodeoxyglucose positron emission tomography (^18^F-FDG PET-CT or PET-CT thereon for simplicity), combined with CT, has become an essential tool for improving the planned target volume delineation of the primary tumor and nodal areas [12]. ^18^F-FDG is a glucose analogue that accumulates in cells with upregulated glucose metabolism, such as cancer cells, allowing PET to detect functional changes before structural abnormalities. As involved field irradiation of the nodal hilar or mediastinal disease is the recommended standard approach as opposed to large mediastinal volume irradiation, PET-CT is critical to encompassing involved areas [3]. Moreover, confirmation of metastatic disease undetectable by simple CTs may drastically change the overall oncological therapeutic plan, including adopting metastatic site irradiation in patients with oligometastatic disease [13].

We aimed to investigate how functional PET-CT imaging affects target volume delineation in RT planning and the overall oncological therapeutic approach in patients with inoperable non-metastatic NSCLC. To achieve this objective, a prospective comparative evaluation of the planning target volume (PTV) delineation between an initial CT-based plan and a corrected plan obtained following the immediate introduction of PET-CT images was performed.

## 2. Materials and Methods

### 2.1. Study Details

We enrolled 34 consecutive patients with histologically diagnosed non-small-cell lung cancer (NSCLC) from April 2023 to February 2025, who were referred for therapy at the Radiotherapy/Oncology Department. The study was conducted in collaboration with the Nuclear Medicine Department of the University Hospital of Alexandroupolis. All histopathologically diagnosed NSCLC patients underwent PET-CT in the repeatable treatment position required for RT planning, lying supine with their arms supported above with a rest device. A flat bed was used to simulate the patient’s exact positioning on the LINAC bed. Planning was performed the day after the PET-CT simulation, and RT started within the following two days.

The inclusion criteria comprised patients with a good performance status (PS), histologically confirmed non-small-cell lung cancer (NSCLC), and no evidence of distant metastases on standard CT imaging, who were scheduled to receive radical chemoradiotherapy followed by anti-PD-1 maintenance one-year immunotherapy. Patients with severe cardiovascular, renal, rheumatic, or psychiatric disease, PS ≥ 2, or history of previous chest irradiation were excluded. These exclusion criteria were primarily applied for ethical reasons, and because most patients who meet the above criteria are often not considered for radical radiotherapy or chemo-RT [14].

The Ethics and Research Committeee of the University Hospital of Alexandroupolis approved the study (No. ES1 12-01-2023). All patients gave written informed consent, which permitted the use of their clinical and laboratory data anonymously for research and publication purposes.

The demographics and clinical data of the participants are presented in Table 1. The age of the participants ranged from 40 to 79, with a mean age of 69.75 years. The mean maximal standardized uptake value (SUV_max_) of lesions was 15.51 ± 11.59 on ^18^F-FDG PET-CT images. The 8^th^ American Joint Committee on Cancer Tumor Nodes Metastasis (AJCC TNM) system was used for lung cancer staging [15].

### 2.2. Immobilization Equipment

The patient was positioned in a supine treatment posture on a carbon fiber couch, with their hands above their head. A broad breast and a firm head/neck rest were also utilized to ensure the patient’s comfort. No four-dimensional (4D) system for breath monitoring was applied, and patients were instructed to breathe normally without taking deep breaths or exhalations.

### 2.3. Staff Training

Adequately trained staff are essential for ensuring the quality assurance and safety of procedures. PET/CT-based RT involves collaboration between the technical and medical staff of the nuclear imaging and radiation oncology departments. In our case, all members were trained on the combined requirements for patient setup and imaging protocols to avoid errors and conflicts. Standard PET/CT patient preparation was applied. In addition, patients were thoroughly informed and prepared for the procedures to secure efficient PET/CT-guided RT simulation.

### 2.4. Patient Simulation

PET/CT examinations were performed on the Discovery^TM^MI PET/CT system (General Electric Healthcare, Waukesha, WI, USA), comprising a 4-ring PET system with LightBurst digital detectors and a 128-slice computed tomography (CT) apparatus.

The CT component was performed with a tube voltage of 120 kV, automatic Smart tube current modulation with a range from 100 to 300 mA, and a slice thickness of 3.75 mm for attenuation correction and anatomical localization. The imaging parameters included a pitch factor of 0.984:1, a speed of 39.37 mm/s, a rotation time of 0.5 s, a maximum Field of View (FOV) of 70 cm, a detector coverage of 40 mm, and a coverage speed of 78.75 mm/s.

The PET images were acquired using a 1.3 min per bed position protocol covering the skull base to mid-thigh. Image reconstruction was conducted using iterative reconstruction algorithms of OSEM (Ordered Subset Expectation Maximization) with time-of-flight (TOF) and point spread function (PSF) correction, employing a 256 × 256 matrix and a Gaussian post-filter for smoothing and sharpening.

The tracer Fluorodeoxyglucose labeled with Fluorine-18 (^18^F-FDG) was administered intravenously at a dose of 2.2–2.5 MBq/kg (0.059–0.067 mCi/kg) by a nuclear physician. Each patient remained in a waiting room for 50–60 min to allow for tracer distribution and uptake in target tissues. The waiting rooms are appropriately shielded to limit radiation exposure to medical staff and other patients.

Before imaging, all patients fasted for at least 4–6 h to maintain low serum glucose levels. Serum glucose levels were measured before radiotracer injection to ensure they were within an acceptable range. Patients were encouraged to stay hydrated with water and avoid intensive physical activity before the scan. Radiation exposure was carefully monitored, and all procedures were conducted under institutional ethical guidelines, with informed consent obtained from all participants.

Respiratory motion was visually assessed during image review to minimize its impact on lesion localization and contouring. Patients were instructed to breathe comfortably and avoid deep breathing motions. Although no formal motion correction algorithm was applied due to the absence of a 4D PET-CT system, experienced staff took care to interpret areas of potential blurring with caution.

System calibration applied in image-based treatment planning to ensure precise dose estimations in radiation therapy. More details referred to Appendix A.

### 2.5. PET-CT Interpretation

An experienced nuclear physician analyzed all PET images. Qualitative assessment was complemented by quantitative evaluation, including SUVmax determination, in all tumor lesions by placing a rectangular region of interest (ROI) that covered the entire volume of each tumor focus. Typically, malignancies were defined as regions of abnormal FDG uptake with standardized uptake values (SUV) of 2.5 or above. Less intense uptake of ^18^F-FDG was also regarded as indicative of a tumor if a corresponding abnormality was observed on CT images.

### 2.6. Radiotherapy Schedule Details

All patients were treated with VMAT with IGRT using a 6 MV Elekta Infinity Linear Accelerator (Elekta, Stockholm, Sweden) equipped with an Agility head (Elekta). The treatment plans were created using Monaco TPS version 6.1.4.0 (Elekta CMS, Maryland Heights, MO, USA). The dose was prescribed to the International Commission on Radiation Units and Measurements (ICRU) reference point for lung cancer. The plans were optimized to maximize the dose of the PTV while minimizing the dose of the surrounding normal tissues. Image-guided radiation therapy (RT) using cone-beam computed tomography (CBCT) was performed before each radiation treatment on the Elekta platform Synergy kV CBCT (XVI) to assess and adjust the patient’s position.

According to the running protocols in our department, two distinct fractionation protocols based on a simultaneous integrated boost technique are applied to offer significant treatment acceleration. The first fractionation schedule includes 14 daily fractions of 3 Gy encompassing the primary tumor and ipsilateral hilar, subcarinal, lower paratracheal, and subaortic lymph nodes [16]. A simultaneous boost of 0.5 Gy is designed to address the primary tumor and radiologically involved nodes (49 Gy total physical dose in 3 weeks). Twenty patients were treated according to this protocol. The second fractionation scheme, suggested by the Royal College of Radiologists in the United Kingdom, involves 20 daily fractions of 2.75 Gy directed to the primary tumor and involved nodes (55 Gy total physical dose in 4 weeks). The non-involved mediastinum receives a daily fraction of 2.5 Gy [17]. Five patients were treated according to this protocol. Five patients refused to undergo prolonged daily RT and received ultra-hypofractionated RT. This scheme involves 5–6 fractions of 6 Gy or 4 fractions of 7–8 Gy, directed to the primary tumor and involved nodes only. Five patients received this schedule. In all RT schedules, lymph nodes were not irradiated in patients with negative PET-CT scans, except for central tumors, where the hilar area was included in the tumor PTV.

In the absence of respiratory-gated PET-CT, motion artifacts were visually evaluated by the interpreting nuclear medicine physicians and radiation oncologists to inform adjustments during PTV delineation. Areas subject to respiratory movement were analyzed across adjacent image slices to enhance localization accuracy.

### 2.7. Chemotherapy Details

All patients received concurrent chemotherapy with a bi-weekly schedule of cisplatin (50 mg/m^2^) and nabPaclitaxel (150 mg/m^2^) for a total of six cycles. Within two weeks of RT completion, patients underwent a CT evaluation to confirm regression or non-progression before starting maintenance immunotherapy with durvalumab, an anti-PDL1 monoclonal antibody (MoAb) (1500 mg flat dose every four weeks).

### 2.8. Radiotherapy Treatment Planning

Each PTV was planned to receive ≥95% and ≤107% of the prescription dose to 95% of its volume. Two separate treatment plans were developed for each patient by an expert radiation oncologist specializing in lung cancer. The first plan was based on CT findings, and immediately afterwards, the radiation oncologist assessed and corrected the CT-based plan after fusion of the CT with the PET image. During this procedure, the radiation oncologist dictated the corrections and the underlying rationale used for these, gradually creating the panel of parameters necessary to interpret the value of PET-CT-based radiotherapy planning. Dose–volume histograms were calculated separately for the PTV and organs at risk (OARs), and the plans received approval for dosimetric analysis.

### 2.9. Target Volume Delineation

Planning CT and PET images were transferred as DICOM images to the Treatment Planning System (TPS) for structure delineation and the production of treatment plans. In the first plan, gross tumor volume (GTV), clinical target volume (CTV), and PTVs were defined exclusively on the anatomical data provided by CT (GTV_CT, CTV_CT, PTV_CT) and in the second plan on images based on fused PET/CT data (GTV_PET-CT, CTV_PET-CT, PTV_PET-CT).

Target volumes and organs at risk (OARs) were contoured according to the European Society for Radiotherapy and Oncology (ESTRO) Advisory Committee on Radiation Oncology Practice (ACROP) guidelines [18]. Following delineation of the primary tumor and involved node GTV, a margin of 0.5 cm was applied to define the CTV. An additional margin of 0.2–0.8 cm (with manual correction according to critical anatomical structure proximity) was used to define the PTV.

The OARs were delineated. Normal lungs were automatically delineated using the Monaco TPS, and both lungs were manually adjusted, minus GTV, excluding the trachea and bronchi. The heart was delineated from the lower edge of the aortic arch to the inferior border of the heart. The spinal cord and the esophagus were also delineated using a 1 cm margin in the superior and inferior radiation fields.

### 2.10. Statistical Analysis

Although the current study aimed to identify and quantify individual changes, the total number of changes suggested by PET-CT to the tumor and nodal PTV was compared to the initial CT-based PTV, and Fisher’s exact test was used to test statistical significance. A *p*-value <0.05 was considered significant.

## 3. Results

### 3.1. Comparison Between RT Plans

The first assessed parameter for comparison related to confirming the absence or revealing the presence of distant metastasis using PET-CT. The overall therapeutic plan was modified to allow patients with extensive metastases, as up-staged by PET-CT, to undergo primary chemo-immunotherapy followed by local palliative radiotherapy (RT), or even subsequent radical chemo-RT in patients who achieved a complete response to the metastatic disease after systemic therapy. However, patients with oligometastatic disease were considered for radical chemo-RT by including the metastatic sites in the RT planning (the dose planned was 3–4 fractions of 7–8 Gy to all metastatic sites). Overall, distant metastases were recorded in the PET-CT of 7/34 (20.6%) patients. Three (3/7) of these patients had oligometastatic disease (one patient with a contralateral lung lesion, and two with bone lesions, one of them with a solitary liver lesion) which was treated radically together with the primary tumor, as described in the methods.

Another important parameter was the underdosing of primary tumor areas of PTV in the CT plan compared to the PET-CT-based plan. Extension of the tumor PTV delineation was necessary in 17/34 (50%) patients. This assessment is crucial to ensure adequate tumor irradiation and minimize any potential risk of recurrence due to underexposure.

Lung atelectasis, with or without pleural effusion, in CT scans significantly complicates the correct identification of tumor areas. In the CT plan, atelectatic lung areas were proven by PET-CT to contain tumoral tissue in 2/34 cases. In 1/34 patients, the primary tumor PTV misdesigned in the CT scan included atelectatic normal lung tissues. PET-CT provides essential guidance for radiation oncologists who, based on CT ambiguity, are called to decide the tumor PTV delineation.

PET-CT significantly helped clarify the status of regional lymph nodes. In 3/34 patients, PET-CT identified lymph node involvement that was not identified on the CT scan. Involved nodes would have been left outside the high-radiation-dose areas. The RT plan was, therefore, changed to include nodal areas. In addition, in 3/34 patients with suspected nodal involvement in the CT scan, PET-CT confirmed the absence of nodal involvement. In these cases, nodal irradiation was omitted from the RT planning, which undoubtedly reduced the overall dose to normal organs.

Identification of metastatic lymph nodes beyond the CT-plan-based RT planning, demanding higher local radiation doses, was documented in 12/34 (35.3%) cases.

The amendments made to the CT plan due to more precise target delineation following CT fusion with PET images are reported in Table 2.

### 3.2. Changes in the Primary Tumor and Nodal Coverage

We further analyzed the changes in the volume (in cc) of the primary tumor and nodal areas included in the respective PTVs between the CT-based and PET-CT-based RT planning.

**Figure 1:**
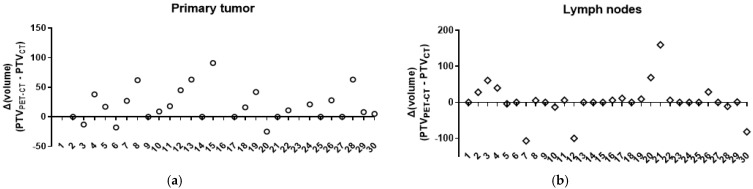
Amendments to the planning target volume (PTV) of the primary tumor and lymph nodes in computed tomography (CT)-based and positron emission tomography (PET)-CT-based planning. (**a**) The individual changes in 30 patients are expressed as the difference between the PET-CT-based and the CT-based primary tumor PTVs. (**b**) The individual changes in 30 patients are expressed as the difference between the PET-CT-based and the CT-based lymph node PTVs.

Out of 30 performed RT plans, a decrease in the primary tumor PTV was noted in 3/30 (10%), with no change in 7/30 (23.3%) and an increase in 20/30 (66.7%) patients (Table 3). Overall, changes to the tumor PTV were made in 23/30 (76.6%) cases (*p* < 0.0001). The individual changes in 30 patients expressed as the difference between the PET-CT-based PTV and the CT-based PTV are shown in Figure 1a.

Out of 30 performed RT plans, a decrease in the lymph node PTV was noted in 6/30 (20%), with no change in 11/30 (36.7%) and an increase in 13/30 (43.3%) patients (Table 3). Overall, changes to the nodal PTV were made in 19/30 (63.3%) cases (*p* < 0.0001). The individual changes in 30 patients expressed as the difference between the PET-CT-based PTV and the CT-based PTV are shown in Figure 1b.

**Figure 2:**
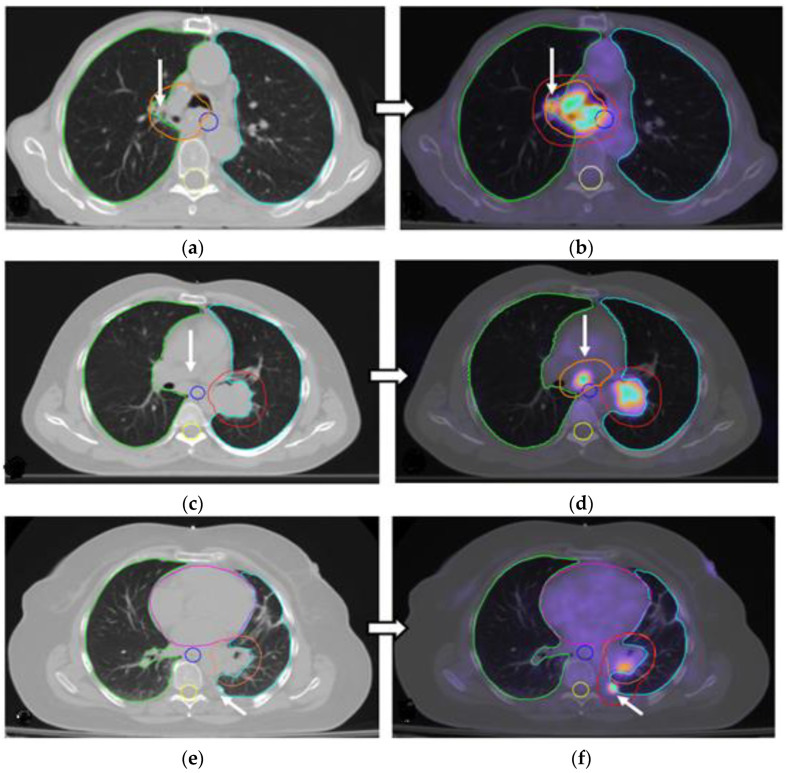

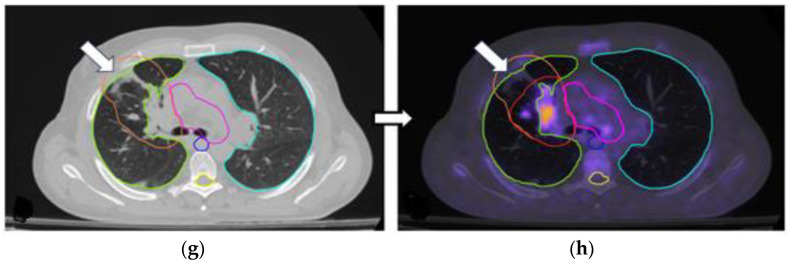
Representative CT (left column) and PET-CT (right column) images showing alterations in planning target volume (PTV) based on PET-CT findings. White arrows highlight PTV alterations. (**a**,**b**) Expansion of the tumor PTV (orange) in PET-CT to include an adjacent metabolically active region (red) not clearly seen on CT. (**c**,**d**) Addition of nodal PTV (orange) in PET-CT to include a previously undetected subcarinal lymph node. (**e**,**f**) PET-CT-guided expansion of the tumor PTV to cover a pleural lesion missed by CT imaging. (**g**,**h**) Reduction in the PTV in PET-CT to spare normal lung tissue previously misclassified as a tumor due to atelectasis. Green and white blue margin shows left and right lungs, corresponding; yellow margin shows spinal cord; blue margin shows trachea and pink margin shows heart based CT (left column) and PET-CT (right column).

Typical examples of changing PTVs during PET-CT-based planning in the primary tumor and involved lymph nodes are illustrated in Figure 2.

## 4. Discussion

Local–regional control (LRC) of lung cancer is crucial for the survival and quality of life of patients with NSCLC [6]. LRC requires accurate tumor delineation, proper RT planning techniques, and dose escalation in target areas, as well as precise radiation dose delivery. VMAT with IGRT planning has been approved as one of the most effective and widely used techniques for treating lung cancer [19]. This technique offers precise dose escalation to the tumor while sparing normal tissue better. The integration of 3D-CBCT real-time imaging during RT sessions enables the correction of position errors or field adjustments, as indicated by monitoring the tumor’s size during therapy, thereby ensuring accurate radiation delivery [20]. ^18^F-FDG PET-CT imaging is a well-established diagnostic tool for NSCLC, with sensitivity ranging from 88% to 96% and specificity ranging from 78% to 92% [21,22]. For this reason, the International Atomic Energy Agency (IAEA), in an expert report from 2006–2007, recognized PET-CT target volume delineation as more accurate than CT target volume delineation, especially for NSCLC [23]. Considering the above notions and data, we investigated the role of PET functional imaging in target volume delineation and radiation therapy (RT) planning for NSCLC.

In CT images provided by a standard CT simulator for NSCLC, the boundary distinction between tumor and normal lung tissues becomes, under certain circumstances, difficult. Atelectasis and obstructive pneumonia are typical examples of uncertainty. PET-CT has been shown to accurately define tumor boundaries, thereby improving local recurrence-free survival (LRC) [24,25]. In our study, the tumor PTV in PET-CT-based planning increased in 20/30 (66.7%) patients and decreased in 3/30 (10%). Some studies also suggest that PET-CT planning allows for changes to the PTV, as it differentiates the tumor from atelectasis [26,27,28]. Our study confirmed this in 3/30 patients with atelectasis or pleural effusion. Regarding mediastinum and chest wall involvement, studies suggest that PET-CT imaging enables the accurate identification of tumor margins near the chest wall and mediastinum, resulting in increased PTV margins in 66% of cases [26,27]. According to Shao et. al., increasing PTVs were indicated by ^18^F-FDG PET-CT images [29].

Irradiation of hilar and mediastinal lymph nodes is also an essential part of RT planning, as modern guidelines suggest avoiding extensive mediastinal irradiation in favor of focal radiation therapy to involved nodes. CT images often underestimate small involved nodes or, conversely, cannot distinguish between inflammatory nodal reactions and nodal invasion. PET-CT confirmed lymph node involvement in an additional 43% of patients compared to CT images, while nodal involvement suggested in CT was proven to be inflammatory in a further 20% of cases. Another interesting finding of our study was the decision to increase the radiation dose, especially in lymph node areas, based on PET-CT images in 35% of cases. Whether increased dose coverage enhances the possibility of better tumor control rates demands further investigation [30].

Ιt is worth mentioning that in the current study, we included details about system calibration procedures and staff training [23,31]. Without standardized calibration protocols, the reliability of PET-CT in tumor delineation volumes and RT dose delivery could be compromised. All technical and medical professionals from the nuclear imaging and radiation oncology departments should be well trained to ensure efficiency and patient safety during procedures. All staff members must be familiar with the combined patient setup and imaging protocols to prevent mistakes and inaccuracies [31].

Although our study included a relatively small patient sample, it was a prospective, single-institution study in which PET-CT was used as a real-time simulator, and the entire procedure took place under the supervision of a physicist and a doctor. Volume delineation was performed by a single radiation oncologist with extensive experience for all patients and subsequently cross-checked by nuclear medicine physicians. To improve the reliability of the research, two physicists standardized the immobilization equipment and system calibration and constructed the treatment plan for every patient in both sets of images. Another limitation of this study is the absence of a respiratory-gated (4D) PET-CT system, the potential of which warrants further investigation. 4D-PET imaging incorporates data from different phases of the respiratory cycle, reducing motion blurring and improving the spatial accuracy of tumor visualization. This can result in more precise delineation of the planning target volume (PTV), especially in tumors that exhibit significant movement with respiration. Guerra et al. demonstrated that such a system could significantly enhance both tumor PTV and lung tissue delineation [32]. Despite the critical RT planning changes suggested by PET-CT and implemented for the treatment of our patients, which was the aim of the current study, survival data are not reported, as the actual value of RT planning optimization is difficult to test outside randomized trials. However, performing randomized trials to compare CT versus PET-CT RT planning raises critical ethical issues, and in our opinion, the necessity and acceptance of the superiority of PET-CT planning can only be established through descriptive studies.

Furthermore, the role of multidisciplinary team (MDT) conferences is increasingly recognized as essential in the management of NSCLC. MDT discussions facilitate the integration of diagnostic imaging, histopathological findings, and clinical assessments, resulting in more accurate staging and optimized treatment strategies. In our study, the collaboration between radiation oncologists and nuclear medicine specialists facilitated more precise PET-CT-based target delineation. Numerous studies have demonstrated that MDT approaches enhance adherence to clinical guidelines, decrease time to treatment, and are linked to improved survival outcomes and quality of life in patients with lung cancer. Incorporating PET-CT findings into these conferences enhances decision-making by offering comprehensive disease characterization and personalized treatment recommendations.

In recent years, Artificial Intelligence (AI) and advanced image processing techniques have shown significant promise in improving the management of NSCLC. AI-driven algorithms are being developed to support automated segmentation of tumors and involved lymph nodes, potentially enhancing the speed and consistency of target volume delineation in radiotherapy planning [33]. Furthermore, radiomics—the extraction of high-dimensional features from imaging data—combined with machine learning approaches, may enable more accurate prognostic stratification and prediction of treatment response. When integrated with PET-CT data, AI models can further refine treatment personalization by identifying subtle metabolic and morphological patterns not easily detectable by human observers. While these technologies are still evolving, their future incorporation into clinical workflows could significantly augment the precision and efficiency of NSCLC care [34,35].

## 5. Conclusions

^18^F-FDG PET-CT-based PTV delineation yields meaningful changes in the RT field areas and dose distribution, as undetectable lymph node involvement, uncertain primary tumor extension, and oligometastatic disease become readily visible, thereby improving RT treatment planning and dose delivery. Tumor underdosing is avoided, and in some patients with atelectasis and pleural effusions, RT fields are appropriately adjusted. Whether this PET-CT-standardized optimization of RT planning also benefits locoregional control and survival requires further investigation in larger series of patients with adequate follow-up.

## Figures and Tables

**Table 1:** Demographics and clinical characteristics of participants.

Feature	
Patients (number)	34
Patient characteristics	
Male	31
Female	3
Age (median ± SD)	69.75 ± 9 years
Height (cm) (median ± SD)	172 ± 6.8 cm
Weight (kg) (median ± SD)	73.5 ± 16.5 kg
Blood glucose	116.03 ± 17.64 mg/dL
Tumor location	
Left upper lobe	15
Left lower lobe	2
Right upper lobe	9
Right middle lobe	5
Right lower lobe	3
Central location	22
Peripheral location	12
Histology	
Adenocarcinoma	11
Squamous cell carcinoma	21
Large-cell carcinoma	2
CT-based TNM staging	
Τ0 Ν2 *	2
Τ2 Ν0 *	2
Τ2Ν3	1
Τ3 Ν0	4
Τ3 Ν1	3
Τ3 Ν2	7
Τ3Ν3	3
Τ4 Ν0	2
Τ4 Ν2	3
Τ4 Ν3	7

* Patients inoperable for medical reasons. Abbreviations: SD, standard deviation; CT, computed tomography; ΤΝΜ, Tumor, Nodes, Metastasis stage.

**Table 2:** Changes in RT planning applied after PET-CT imaging introduction for target delineation.

Parameter	Number of Patients Requiring Therapeutic Plan Modification	Number of Patients Requiring RT Plan Modification
Identification of extensive distant metastasis	4/34 (11.8%)	(*)
Detection of oligometastatic disease included in the RT planning	-	3/34 (8.8%)
Inadequate coverage of the primary tumor area	-	17/34 (50%)
Detection of lymph node involvement in cases with negative CT findings	-	3/34 (8.8%)
Exclusion of lymph node involvement in cases with suspected CT findings	-	3/34 (8.8%)
Identification of additional involved nodes demanding larger RT fields and/or higher RT doses.	-	12/34 (35.3%)
Accurate identification of the primary tumor location in patients with pleural effusion and/or lung atelectasis	-	3/34 (8.8%)

(*) Patients were treated with systemic therapy and palliative RT.

**Table 3:** Amendments to the primary tumor PTV.

Primary Tumor PTV	Number of pts (%)	Median Change/Range—*p*-Value
Decrease	3/30 (10%)	−18 (−25 to −13)
Stable	7/30 (23.3%)	0 (0)
Increase	20/30 (66.7%)	+33 (5 to 581)
		*p* < 0.0001
Lymph node PTV *	No of pts (%)	Median change/range—*p*-value
Decrease	6/30 (20%)	−47.5 (−107 to −4)
Stable	11/30 (36.7%)	0 (0)
Increase	13/30 (43.3%)	11 (1 to 160)
		*p* < 0.0001

* Abbreviations: PTV, planning target volume; No, number.

**Figure A1:**
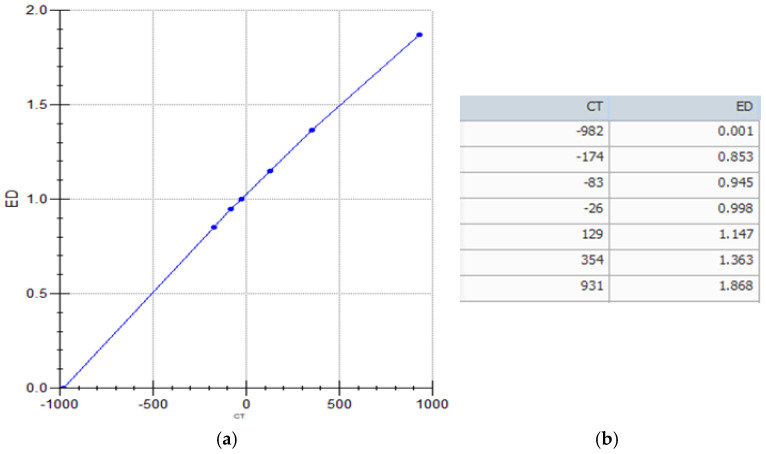
(**a**) Computed tomography-to-electron density (CT-to-ED) curve and (**b**) data which were imported into our Treatment Planning System (TPS).

## Data Availability

Data is unavailable due to privacy or ethical restrictions.

## Abbreviations

The following abbreviations are used in this manuscript:

IARC	International Agency for Research on Cancer
NSCLC	Non-Small-Cell Lung Carcinoma
SCLC	Small-Cell Lung Carcinoma
NCCN	National Comprehensive Cancer Network
IASLC	International Association for the Study of Lung Cancer
LRC	Locoregional Control
RT	Radiotherapy
VMAT	Volumetric Modulated Arc Therapy
IGRT	Image-Guided Radiation Therapy
LINAC	Linear Accelerator
CT	Computed Tomography
^18^F-FDG PET-CT or PET-CT	^18^Fluorine-Labeled Fluorodeoxyglucose Positron Emission Tomography
PTV	Planning Target Volume
PS	Performance Status
AJCC TNM	American Joint Committee on Cancer—Tumor, Nodes, Metastasis
4D	Four-Dimensional
FOV	Field of View
OSEM	Ordered Subset Expectation Maximization
TOF	Time-of-Flight
PSF	Point Spread Function
SUVmax	Maximal Standardized Uptake Value
ROI	Region of Interest
SUV	Standardized Uptake Values
^18^F-FDG	Fluorodeoxyglucose Labeled with Fluorine-18
ICRU	International Commission on Radiation Units and Measurements
CBCT	Cone-Beam Computed Tomography
OARs	Organs at Risk
TPS	Treatment Planning System
GTV	Gross Tumor Volume
CTV	Clinical Target Volume
ESTRO	European Society for Radiotherapy and Oncology
ACROP	Advisory Committee on Radiation Oncology Practice

## Appendix A. System Calibration

Using tissue inhomogeneity correction in image-based treatment planning improves the accuracy of radiation dose calculations. The computed tomography-to-electron density (CT-to-ED) curve ensures precise dose estimations in radiation therapy. The Catphan 503 phantom, which has many tissue-equivalent inserts that replicate diverse electron densities, including lung, muscle, and bone, is often utilized to calibrate this curve in PET/CT-based planning. Following the patient imaging technique, the calibration process begins with a CT scan of the phantom. After being acquired, the Hounsfield Unit (HU) values for the material inserts are calculated and plotted against their known electron densities to create a conversion curve. This calibration is necessary for heterogeneity adjustments, which enable the Treatment Planning System (TPS) to adjust for tissue variances and improve radiation dose distribution accuracy. By including the CT-to-ED curve in the TPS, clinicians can increase the accuracy of PET/CT-guided radiation, resulting in more effective and individualized treatment procedures.

Figure A1 illustrates the Catphan 503 phantom and computed tomography-to-electron density (CT-to-ED) curve which was used in our clinic Treatment Planning System (TPS).
